# Supplementary material for: Cross-sectional survey of child weight management service provision by acute NHS trusts across England in 2020/2021
Source: BMJ Open. 2022 Nov 10;12(11):e061971. doi: 10.1136/bmjopen-2022-061971 (PMC9670955; doi:10.1136/bmjopen-2022-061971)
Supplement: Supplementary data [file bmjopen-2022-061971supp001.pdf]

Supplementary File 1

Survey

Under the provisions of the Freedom of Information Act 2000, we request the following information:

What is the name of your acute trust?Click or tap here to enter text.

(1) Does your trust provide a weight management service for children living with obesity?

Yes ☐ No ☐

If No, you need not answer further.

If yes, what is the name of the service and where delivered:Click or tap here to enter text.

Who leads this service?Click or tap here to enter text.

It is a hospital ☐ or community-based☐ clinic

(2) What if any are the criteria for eligibility to attend (for instance BMI >98th percentile)?

None ☐

Eligible if: Click or tap here to enter text.

(3) Through what mechanism is the service funded?

CCG	☐
NHS England	☐
Research	☐
Charity	☐
Trust Self-Funding	☐
Other	☐

Please specify Click or tap here to enter text.

(4) Who are the key personnel providing service? Please tick all who apply:

a.	Paediatricians	☐
b.	Dieticians	☐
c.	Psychologists	☐
d.	Trained volunteers	☐
e.	Hospital or Community based Nursing staff	☐
f.	Exercise specialists	☐
g.	Social workers	☐
h.	CAHMS	☐
i.	Youth workers	☐

(5) How many new cases are seen each year? Click or tap here to enter text.

(6) Over how many weeks is the clinic intervention delivered? Click or tap here to enter text.

(7) What is the usual follow up period (entry to discharge?) Click or tap here to enter text.

(8) What final outcomes are collected: Examples: change in BMI, BMI SDS, Self Esteem, Waist Circumference, recruitment, retention.

Please list all. Click or tap here to enter text.

Many thanks for the information you provide.

Professor Julian Hamilton-Shield, University of Bristol

Supplementary File 2

Supplementary Table 1: Service provision according to catchment area characteristics of acute NHS trust

	Mean % of patients within catchment area of acute NHS trusts according to ethnicity and service provision					Mean IMD score of acute NHS trusts
	White	Black	Asian	Mixed	Other	
Service provided	85.5	3.9	7.3	2.2	1.1	22.8
Service not provided	88.6	2.4	6.3	1.9	0.7	20.8
Did not respond to survey	81.7	4.7	9.7	2.6	1.3	22.4
Ethnicity was available for 142 trusts and IMD score for 127 trusts						
