# Supplementary material for: Cross-sectional survey of child weight management service provision by acute NHS trusts across England in 2020/2021
Source: BMJ Open. 2022 Nov 10;12(11):e061971. doi: 10.1136/bmjopen-2022-061971 (PMC9670955; doi:10.1136/bmjopen-2022-061971)
Supplement: Author's manuscript [file bmjopen-2022-061971.draft_revisions.pdf]

BMJ Open is committed to open peer review. As part of this commitment we make the peer review history of every article we publish publicly available.

When an article is published we post the peer reviewers' comments and the authors' responses online. We also post the versions of the paper that were used during peer review. These are the versions that the peer review comments apply to.

The versions of the paper that follow are the versions that were submitted during the peer review process. They are not the versions of record or the final published versions. They should not be cited or distributed as the published version of this manuscript.

BMJ Open is an open access journal and the full, final, typeset and author-corrected version of record of the manuscript is available on our site with no access controls, subscription charges or pay-per-view fees (http://bmjopen.bmj.com).

If you have any questions on BMJ Open's open peer review process please email info.bmjopen@bmj.com

BMJ Open

A cross-sectional survey of child weight management service provision by acute NHS trusts across England in 2020

Journal:	*BMJ Open*
Manuscript ID	bmjopen-2022-061971
Article Type:	Original research
Date Submitted by the Author:	14-Feb-2022

Complete List of Authors:	Mears, Ruth; University of Bristol, Centre for Academic Primary Care, Bristol Medical School; University of Bristol, Centre for Exercise Nutrition and Health Sciences<br>Leadbetter, S; University Hospitals Bristol and Weston NHS Foundation Trust, National Institute for Health Research Bristol Biomedical Research Centre; University of Bristol, Faculty of Health Sciences<br>Candler, Toby; University Hospitals Bristol and Weston NHS Foundation Trust, National Institute for Health Research Bristol Biomedical Research Centre; University of Bristol, Faculty of Health Sciences<br>Sutton, Hannah ; University of Bristol, Faculty of Health Sciences<br>Sharp, Deborah; University of Bristol, Centre for Academic Primary Care, Bristol Medical School<br>Shield, Julian; University Hospitals Bristol and Weston NHS Foundation Trust, National Institute for Health Research Bristol Biomedical Research Centre

Keywords:	PUBLIC HEALTH, PAEDIATRICS, Health policy < HEALTH SERVICES ADMINISTRATION & MANAGEMENT, Organisation of health services < HEALTH SERVICES ADMINISTRATION & MANAGEMENT

SCHOLARONE™ Manuscripts

I, the Submitting Author has the right to grant and does grant on behalf of all authors of the Work (as defined in the below author licence), an exclusive licence and/or a non-exclusive licence for contributions from authors who are: i) UK Crown employees; ii) where BMJ has agreed a CC-BY licence shall apply, and/or iii) in accordance with the terms applicable for US Federal Government officers or employees acting as part of their official duties; on a worldwide, perpetual, irrevocable, royalty-free basis to BMJ Publishing Group Ltd ("BMJ") its licensees and where the relevant Journal is co-owned by BMJ to the co-owners of the Journal, to publish the Work in this journal and any other BMJ products and to exploit all rights, as set out in our licence.

The Submitting Author accepts and understands that any supply made under these terms is made by BMJ to the Submitting Author unless you are acting as an employee on behalf of your employer or a postgraduate student of an affiliated institution which is paying any applicable article publishing charge ("APC") for Open Access articles. Where the Submitting Author wishes to make the Work available on an Open Access basis (and intends to pay the relevant APC), the terms of reuse of such Open Access shall be governed by a Creative Commons licence – details of these licences and which Creative Commons licence will apply to this Work are set out in our licence referred to above.

Other than as permitted in any relevant BMJ Author's Self Archiving Policies, I confirm this Work has not been accepted for publication elsewhere, is not being considered for publication elsewhere and does not duplicate material already published. I confirm all authors consent to publication of this Work and authorise the granting of this licence.

For peer review only

A cross-sectional survey of child weight management service provision by acute NHS trusts across England in 2020

Mears R [1,2], Leadbetter S [3,4], Candler T [3,4], Sutton H [4], Sharp D [1], Hamilton-Shield J [3,4].

Centre for Academic Primary Care, Bristol Medical School, University of Bristol, BS8 2PS, UK [1]. Centre for Exercise Nutrition and Health Sciences, School for Policy Studies, University of Bristol, BS8 1TZ, UK [2]. NIHR Bristol Biomedical Research Centre, Nutrition Theme, University of Bristol, BS1 2NT, UK [3]. Faculty of Health Sciences, University of Bristol, BS2 8DZ, UK [4].

Corresponding Author: Dr Ruth Mears, rm14101@bristol.ac.uk

Word Count: 2202

For peer review only

Abstract

Objective: With one in five children in England living with obesity, we mapped the geographical distribution and format of child weight management services provided by acute NHS trusts across England, to identify breadth of service provision.

Design: A cross-sectional survey.

Setting: The survey was sent to all acute NHS trusts (n=148) in England in 2020.

Participants: Responses were received from 139 of 148 (94%) acute NHS trusts.

Outcome Measures: The survey asked each acute NHS trust whether they provide a weight management service for children living with obesity. For those trusts providing a service, data were collected on eligibility criteria, funding source, personnel involved, number of new patients seen per year, intervention duration, follow-up length and outcome measures. Service characteristics were reported using descriptive statistics. Service provision was analysed in the context of ethnicity and IMD score of the trust catchment area.

Results*:* From the 139 survey respondents, 23% stated that they provided a weight management service for children living with obesity. There were inequalities in the proportion of acute NHS trusts providing a service across the different regions of England, ranging from 4% (Midlands) to 36% (London). For trusts providing a service, there was variability in the number of new cases seen per year, eligibility criteria, funding source, intervention format and outcome measures collected. A multi-disciplinary approach was not routinely provided, with only 41% of services reporting ≥3 different staff disciplines.

Conclusions: In 2020, there were geographical inequalities in weight management service provision by acute NHS trusts for children living with obesity. Services provided lacked standardisation, did not routinely offer children multidisciplinary care and were insufficient in size to meet need.

For peer review only

Strengths and Limitations

- There are no previous studies in the research literature exploring the geographical equity and format of child weight management services provided by acute NHS trusts across England.
- The survey response rate (94%) was good, allowing for an accurate oversight of service provision by acute NHS trusts in England.
- Outcome data for services were not collected, meaning service effectiveness could not be assessed.
- Ethnicity and measures of deprivation data for the individuals accessing the services within each acute NHS trust were not collected, thereby limiting ethnicity and deprivation comparisons to acute NHS trust catchment area level data.

For peer review only

Introduction

Obesity in childhood and adolescence represents a major public health challenge with short and long-term consequences at both an individual and societal level. Children with obesity are more likely to have type 2 diabetes, dyslipidaemia, hypertension, metabolic syndrome, non-alcoholic fatty liver disease and obstructive sleep apnoea [1 2]. Around 55% of children with obesity will remain obese as adolescents, and 80% of adolescents with obesity will continue to be obese as adults [3]. Obesity has both direct healthcare related costs and indirect costs to society through short and long-term inability to work [4 5]. In 2018, obesity cost the UK an estimated £60 billion (3% of its GDP) [6 7].

In the UK, child weight management services are defined as: tier 1 (obesity prevention), tier 2 (lifestyle weight management services), tier 3 (specialist obesity services) and tier 4 (pharmacological or surgical obesity services) [8]. Tier 1 and tier 2 services are commissioned by Public Health England (now likely to reside in the Office for Health Promotion) working nationally and within local teams. In theory, tier 3 and tier 4 services cater for children with severe or complex obesity, or special needs, and are commissioned by Clinical Commissioning Groups (CCGs) working within the National Health Service (NHS). There is no mandatory requirement for PHE or NHS to commission child weight management services.

Commissioning guidance for specialist services for children with severe or complex obesity, recommend a multi-disciplinary team (MDT) approach, including a paediatrician, dietitian, nurse, physical therapist, psychologist, liaison psychiatrist and MDT access to a social worker [9]. Previous studies have explored the nature and provision of tier 2 weight management services across England, but there is no documentation within the research literature to gain national oversight of specialist obesity weight management services provided by acute NHS trusts in England [10 11]. A Public Health England (PHE) mapping study of tier 3 weight management services for children in 2015 reported a poor response rate limiting accurate representation of service provision at the time [11]. The lack of understanding about current provision of specialist obesity services was highlighted in the Chief Medical Officer's (CMO) report on childhood obesity [7].

The primary objective of the study was to determine how many acute NHS trusts provide a weight management service for children, and the geographical distribution of these

For peer review only

services. The secondary objective of the study was to understand the nature of weight management services provided by acute NHS trusts, in terms of eligibility criteria, funding source, whether a MDT approach operated, the reach of the service and details regarding the intervention and outcome data collected. The final objective was to understand whether there were any inequalities in ethnicity and deprivation scores between the catchment populations of NHS trusts providing a weight management service, compared to those that did not.

Methods

Design of the survey

The survey comprised eight questions and was developed by JHS based on his experience working within a paediatric obesity specialist clinic at the Bristol Royal Hospital for Children (BRHC), University Hospitals Bristol & Weston (UHBW) NHS Foundation Trust (Supplementary File 1). All acute NHS trusts in England were asked, 'Does your trust provide a weight management service for children living with obesity?'. Those affirming they did, were asked questions regarding the service's eligibility criteria, source of funding, key personnel involved, number of new patients seen per year, duration of their intervention, length of follow up and outcome data collected by their service. Questions were either free-text or multiple choice (single or multiple answer questions).

Recruitment and characteristics of acute NHS trust participants

The survey was sent as a Freedom of Information (FOI) request to 148 acute NHS trusts across England. Where a response was not received by the acute NHS trust within 20 working days, a further reminder email was sent. If no response was received, JHS contacted potential service leads from a list provided by the Children and Young People (CYP) Transformation Programme Team at NHS England.

The 'PHE NHS acute trust catchment population tool' was used to obtain data (where available) about the catchment population for each acute NHS trust, in terms of Index of Multiple Deprivation (IMD) score and ethnicity [12]. This tool also provided details regarding the acute NHS trust type (small, medium, large, multi-service, specialist and teaching).

For peer review only

Data analysis

Data were collated and analysed in Excel and Stata. In order to understand potential inequalities in the geographical distribution of weight management services for children, trusts were organised into the seven regions of England (North East and Yorkshire, North West, South East, South West, London, East of England, and Midlands) and the proportion of trusts within each geographical region providing a service was quantified. The mean IMD score and mean proportion of patients within each different ethnicity category were compared between those trusts providing and not providing a service. Comparisons between the proportion of trusts offering and not offering a service were also made according to acute NHS trust type. For acute NHS trusts providing a weight management service, the characteristics of the service provided were reported using descriptive statistics.

Patient and public involvement

Patients or the public were not involved in the design, or conduct, or reporting, or dissemination plans of our research.

Ethical approval

This is a health services research paper. The survey was sent to acute NHS trusts as a FOI request, therefore Ethics Committee review was not required.

Results

Responses were received from 139 of 148 acute NHS trusts contacted (94%). From the 139 respondents, 32 trusts (23%) stated that they provide a weight management service for children living with obesity. Some trusts offered more than one service for children living with obesity. The mean IMD score, and ethnicity of patients were similar between those within a catchment area of an acute NHS trust providing and not providing a weight management service (Supplementary File 2).

Location in England and acute NHS trust type

The greatest proportion of trusts providing a weight management service were within London (36% of trusts). In contrast, only 4% of trusts in the Midlands provided a service

For peer review only

(Table 1). Multi-service and teaching trusts were more likely to provide a weight management service than other acute NHS trusts (Table 2).

Table 1: Geographical distribution of services across England

	Service provided (n=number of acute NHS trusts)	No service provided (n=number of acute NHS trusts)	No response to survey (n=number of acute NHS trusts)
North East and Yorkshire	7 (31.8%)	15 (68.2%)	0 (0%)
North West	4 (17.4%)	17 (73.9%)	2 (8.7%)
South East	4 (21.1%)	15 (78.9%)	0 (0%)
London	9 (36.0%)	14 (56%)	2 (8%)
East of England	3 (16.7%)	14 (77.8%)	1 (5.6%)
Midlands	1 (4.2%)	20 (83.3%)	3 (12.5%)
South West	4 (23.5%)	12 (70.6%)	1 (5.9%)

Table 2: Service provision according to acute NHS trust type

	Service provided (n=number of acute NHS trusts)	No service provided (n=number of acute NHS trusts)	No response to survey (n=number of acute NHS trusts)
Acute – Small	6 (19.4%)	24 (77.4%)	1 (3.2%)
Acute – Medium	6 (19.4%)	23 (74.2%)	2 (6.5%)
Acute – Large	5 (18.5%)	20 (74.1%)	2 (7.4%)
Acute - Multi-Service	2 (66.7%)	1 (33.3%)	0 (0%)
Acute – Specialist	3 (18.8%)	13 (81.3%)	0 (0%)
Acute – Teaching	10 (29.4%)	22 (64.7%)	2 (5.9%)
Data regarding the type of acute NHS trust was available for 142 trusts			

Site of intervention, eligibility criteria, funding source and staffing

The characteristics of weight management services provided by acute NHS trusts across England, is summarised in Table 3. Over half of weight management services were hospital based only (59%). There was considerable variation in the source of funding for these services, with 26% of trusts receiving funding from multiple sources. CCGs were the most frequently cited source of funding.

For peer review only

Eligibility criteria for services were mainly based around a measure of weight (e.g., Body Mass Index [BMI], BMI percentile, BMI z-score) but some services reported less specific eligibility criteria (e.g. 'all referrals where a dietetic or nutrition related problem has been identified by a medic'). Dietitians were the most frequently reported personnel involved in service provision, followed by paediatricians. Most services did not provide a multi-disciplinary team approach, with 31% of services reporting only one type of staffing personnel involved.

Table 3: Characteristics of weight management services provided by acute NHS trusts

	**Number of acute NHS trusts (n=32)**
**Location of services provided by each acute NHS trust**	
Hospital	19
Community	8
Hospital and community-based	4
Not specified	1
**Source of Funding ***	
Clinical Commissioning Group (CCG)	15
Trust Self-Funding	9
NHS England	2
County Council, Local Authority or Public Health	7
Other	7
Not specified	1
**Eligibility criteria ****	
BMI >91st centile	5
BMI >91st centile and another criterion	5
BMI >98th centile	5
BMI >98th centile and another criterion	9
BMI >99.6th centile	4
BMI >99.6th centile and another criterion	5
BMI Standard Deviation Score (SDS) >2.2	1
BMI ≥3.33sd in child over 2 years old	1
BMI>+3.5SDS or BMI> +3.0SDS with comorbidities	2
Other	18
**Staff disciplines involved in service provision for each acute NHS trust *****	
Dietitians	26
Paediatricians	16
Hospital or community-based nursing staff	11

Psychologists	6
Exercise specialists	5
Children and Adolescent Mental Health Services (CAMHS)	3
Social workers	2
Not specified	2
Trained volunteers	1
**Number of staff disciplines providing service for each acute NHS trust**	
One	10
Two	7
Three	6
Four	5
Five	1
Six	1
Not specified	2
* Some acute NHS trusts received funding from more than one source for their service(s). ** Some acute NHS trusts reported more than one different eligibility criteria for their service(s). *** Some acute NHS trusts reported more than one different staff disciplines involved in their service provision.	

Characteristics of services provided by acute NHS trusts

Table 4 summarises details of the interventions provided by trusts. There was variation in the number of new cases seen per year, with 61% of acute NHS trusts reporting that their service(s) saw <100 new cases per year. Many trusts reported that the intervention and follow-up period were tailored to the individuals needs rather than following a pre-specified pattern. Outcome measures most frequently collected were BMI, BMI z-score / SDS or BMI centile. A variety of other outcome measures were reported, including a parent or user satisfaction survey, changes to emotional eating behaviours, reduction of inequalities among targeted groups and SCORE-15 index of family functioning and change [13].

Table 4: Details of the services provided by acute NHS trusts

	**Number of acute NHS trusts (n=32)**
**Total number of new cases seen per year for each acute NHS trust**	
<50	11
50-100	8

For peer review only

100-150	4
150-200	2
200-250	0
250-300	0
300-350	2
Not specified	5
**Length of intervention***	
0-6 months	9
6-12 months	4
12-18 months	1
Tailored to the individual	11
Not clearly specified	10
Other	2
**Usual follow-up period***	
0-6 months	6
6-12 months	5
12-18 months	6
over 18 months	4
Tailored to the individual	9
Not clearly specified	3
Other	1
**Outcome Measures**	
BMI	19
BMI-SDS or BMI z-score	11
BMI-centile	2
Weight	7
Waist circumference	2
Body composition	3
Blood pressure	2
Sleep study results	3
Blood tests	4
Imaging	1
Changes to diet	7
Changes to physical activity levels	4
Changes to mental wellbeing	7
Quality of life	2
Other	12

**Respondents sometimes detailed more than one clinical service in the trust so n responses may be greater than 32.*

Discussion

Main Findings

There were significant gaps in the provision of child weight management services by acute NHS trusts across England, with 77% of survey respondents not providing a service. Within England, there was considerable variation in the distribution of services with a greater proportion of acute NHS trusts providing a service in certain geographical regions (e.g., London 36%, and the North-East and Yorkshire 32%) compared to others (e.g., Midlands 4%). There was a lack of consistency in the funding source and eligibility criteria for children to access services between different acute NHS trusts. A multi-disciplinary approach was not routinely provided and there was no standardisation of the service provided between different trusts in terms of length of intervention, follow-up period and outcome measures.

Strengths and Weaknesses

This is the first study in the research literature to explore the nature of child weight management service provision by acute NHS trusts across England. The response rate by acute NHS trusts was good (94%). As acute NHS trusts are typically responsible for the provision of tier 3 weight management services, this paper provides a good indicator of what services are available for children with severe obesity. It was not within the remit of this study to capture non-NHS tier 2 weight management programmes. It was also not possible to merge the data on NHS provision with pre-existing datasets about non-NHS tier 2 weight management provision (i.e. those commissioned by Local Authorities), partly due to the rapid commissioning and de-commissioning of the latter [10] .

Data detailing intervention components and outcome measures were not requested to reduce burden on individual Trust's FOI response teams and improve overall response rate [14]. Thus, it was not possible to assess the relative effectiveness of different interventions offered by each acute NHS trust. However, given the lack of standardization in eligibility criteria and outcome measures across different NHS trusts, meaningful comparative analyses are unlikely to have been possible. Deprivation and ethnicity data for those actually accessing each service were also not collected so comparisons in these measures were limited to acute NHS trust population catchment area data.

For peer review only

Data were collected through a FOI request meaning it is not possible to ascertain who supplied survey data for each NHS trust. The assumption is that the FOI request would have been appropriately handed to the lead for the clinical service.

Contextualisation and Implications of Findings

A lack of adequate multidisciplinary weight management clinics for children with obesity and unequal geographical distribution of available services, has also been described in Australia [15 16]. This contrasts with current guidelines and expert recommendations for obesity management in children, which advises a multidisciplinary team approach to ensure optimal care [9 17 18]. Similar to our study, data reported from Canadian paediatric weight management services has shown variability in eligibility criteria, clinic size and intervention length [19].

In England (2020/21), 14% of children aged 4-5 years old and 26% of children aged 10-11 years old were identified as obese by the National Child Measurement Programme (NCMP). Therefore, the current 32 sparsely distributed acute NHS trust services in England (generally seeing <200 new cases per year), will only reach a minute fraction of obese (or severely obese) children.

A pathway modelling study of National Institute for Health and Care Excellence (NICE) guidance against Health Survey for England data, suggested that there is a 'need for consistent evidence-based commissioning of services across the childhood obesity pathway based on population burden', however our study indicates this is still not happening [20]. Despite the West Midlands having the highest prevalence of severe obesity according to 2019/2020 reception NCMP data (3.1% vs England average of 2.5%), the Midlands region had the lowest proportion of NHS trusts providing a weight management service (n=1/24, 4.2%) [21].

The NHS Long Term Plan aims to treat a further 1000 children a year for severe complications related to their obesity through 'complications of excess weight' (CEW) clinics. These CEW clinics will also have a universal data collection system for patient demographics and outcomes to allow comparison across sites and understand the reach of the service according to ethnicity and deprivation. However with previous estimates from 2014 suggesting that 556,000 children would be eligible for assessment in secondary care,

For peer review only

this planned expansion of services will still not reach the majority of children who may require treatment [20 22].

The lack of consistent sources of funding for specialist obesity clinics in acute NHS trusts likely contributes to the inequalities in the geographical distribution of services. Clearly defined routes of funding for child weight management clinics in acute NHS trusts are needed to help minimise inequalities in service provision. Furthermore, given the 13 percentage-point gap in obesity rates between the most deprived and least deprived children in 2019, services need to ensure that within the geographical areas they provide for, they are reaching the most deprived children [23].

Conclusion

There needs to be a clear realistic national strategy outlining who should receive initial priority for specialist multi-disciplinary obesity care, as the need for support will likely exceed capacity for the foreseeable future and service provision across England should be equitable. The CEW clinics may help to reduce current inequalities in service provision and target those at greatest risk from obesity. However, significant gaps in support for children who are obese but do not meet the CEW clinic criteria will likely remain, especially given the variable tier 2 service provision across England [10] . Furthermore, as referral into the CEW clinic will be reliant on the presence of co-morbidities, consideration needs to be given to determine the most appropriate way of ensuring that children with obesity (or at least severe obesity) are assessed for co-morbidities and thereby given the opportunity to access specialist care where needed.

Funding: Dr Ruth Mears is funded by a National Institute for Health Research (NIHR) In-Practice Fellowship Award (NIHR-IPF-16-10-07) for this research. The NIHR Biomedical Research Centre at University Hospitals Bristol and Weston NHS Foundation Trust supports Professor Julian Hamilton-Shield.

For peer review only

Disclaimer: This publication presents independent research funded by the National Institute for Health Research (NIHR). The views expressed in this publication are those of the authors and not necessarily those of the NIHR.

Competing Interests: JHS is clinical lead for a Tier 3 childhood obesity service in Bristol and will be a hub for the new 'Complications related to excess weight' (CEW) clinics funded by NHS England.

Contributors: JHS designed the study. TC, SL and HC collected the data. RM and JHS analysed the data and drafted the initial manuscript. JHS, TC, SL, HC and DS revised the final manuscript.

Data sharing: All data relevant to the study are included in the article or uploaded as supplementary information.

For peer review only

References

1. Sharma V, Coleman S, Nixon J, et al. A systematic review and meta-analysis estimating the population prevalence of comorbidities in children and adolescents aged 5 to 18 years. *Obes Rev* 2019;20(10):1341-49. doi: 10.1111/obr.12904 [published Online First: 2019/07/26]
2. Li AM, So HK, Au CT, et al. Epidemiology of obstructive sleep apnoea syndrome in Chinese children: a two-phase community study. *Thorax* 2010;65(11):991-7. doi: 10.1136/thx.2010.134858 [published Online First: 2010/10/23]
3. Simmonds M, Llewellyn A, Owen CG, et al. Predicting adult obesity from childhood obesity: a systematic review and meta-analysis. *Obes Rev* 2016;17(2):95-107. doi: 10.1111/obr.12334 [published Online First: 2015/12/24]
4. Wang YC, McPherson K, Marsh T, et al. Health and economic burden of the projected obesity trends in the USA and the UK. *Lancet* 2011;378(9793):815-25. doi: 10.1016/S0140-6736(11)60814-3 [published Online First: 2011/08/30]
5. Goettler A, Grosse A, Sonntag D. Productivity loss due to overweight and obesity: a systematic review of indirect costs. *BMJ Open* 2017;7(10):e014632. doi: 10.1136/bmjopen-2016-014632
6. McKinsey Global Institute. Overcoming obesity: An initial economic analysis.: McKinsey Global Institute, 2014.
7. Davies PDS. Time to Solve Childhood Obesity. An Independent Report by the Chief Medical Officer, 2019.

8. NICE. Weight management: lifestyle services for overweight or obese children and young people. PH47 https://www.nice.org.uk/guidance/ph53 [Accessed August 2021] .

9. Welbourn R, Hopkins J, Dixon JB, et al. Commissioning guidance for weight assessment and management in adults and children with severe complex obesity. *Obesity Reviews* 2018;19(1):14-27. doi: 10.1111/obr.12601

10. Mears R, Jago R, Sharp D, et al. Exploring how lifestyle weight management programmes for children are commissioned and evaluated in England: a mixed methodology study. *BMJ Open* 2019;9(12):e025423. doi: 10.1136/bmjopen-2018-025423 [published Online First: 2019/12/19]

11. Public Health England (PHE). National mapping of weight management services. Provision of Tier 2 and Tier 3 services in England: Public Health England, 2015.

12. Public Health England (PHE). PHE NHS Acute Trust Catchment Populations Tool https://app.powerbi.com/view?r=eyJrIjoiODZmNGQ0YzItZDAwZi00MzFiLWE4NzAtMzVmNTUwMThmMTVlIiwidCI6ImVlNGUxNDk5LTRhMzUtNGIyZS1hZDQ3LTVmM2NmOWRlODY2NiIsImMiOjh92020 [Accessed October 2021]

13. Stratton P, Lask J, Bland J, et al. Detecting therapeutic improvement early in therapy: Validation of the SCORE-15 index of family functioning and change. *Journal of Family Therapy* 2014;36(1):3-19. doi: 10.1111/1467-6427.12022

14. UCL. Making Freedom of Information Requests: A Guide for Academic Researchers. The Constitution Unit 2012 https://www.ucl.ac.uk/constitution-unit/sites/constitution_unit/files/academics-guide-to-foi.pdf. [Accessed February 2022].

15. McMaster CM, Calleja E, Cohen J, et al. Current status of multi-disciplinary paediatric weight management services in Australia. *J Paediatr Child Health* 2021;57(8):1259-66. doi: 10.1111/jpc.15439 [published Online First: 2021/03/17]

16. Spilchak PJ, Denney-Wilson E, King L, et al. Tertiary paediatric obesity services in Australia. *J Paediatr Child Health* 2008;44(5):243-7. doi: 10.1111/j.1440-1754.2007.01266.x [published Online First: 2008/01/16]

17. Styne DM, Arslanian SA, Connor EL, et al. Pediatric Obesity-Assessment, Treatment, and Prevention: An Endocrine Society Clinical Practice Guideline. *J Clin Endocrinol Metab* 2017;102(3):709-57. doi: 10.1210/jc.2016-2573 [published Online First: 2017/03/31]

18. Barlow SE. Expert committee recommendations regarding the prevention, assessment, and treatment of child and adolescent overweight and obesity: summary report. *Pediatrics* 2007;120 Suppl 4:S164-92. doi: 10.1542/peds.2007-2329C [published Online First: 2007/12/18]

For peer review only

19. Zahn K, Ibrahim Q, Ball GDC, et al. Variability in How Canadian Pediatric Weight Management Clinics Deliver Care: Evidence from the CANadian Pediatric Weight Management Registry. *Child Obes* 2021;17(6):420-26. doi: 10.1089/chi.2021.0011 [published Online First: 2021/05/13]

20. Viner RM, Kinra S, Nicholls D, et al. Burden of child and adolescent obesity on health services in England. *Arch Dis Child* 2018;103(3):247-54. doi: 10.1136/archdischild-2017-313009 [published Online First: 2017/08/03]

21.Public Health England (PHE). Obesity Profile PHE Fingertips NCMP data https://fingertips.phe.org.uk/profile/national-child-measurement-programme/data#page/0/gid/8000011/ati/6/iid/90316/age/200/sex/4/cat/-1/ctp/-1/cid/4/tbm/1 [Accessed August 2021]

22. NHS. NHS Long Term Plan. Chapter 2: More NHS action on prevention and health inequalities

23. Holmes J. Tackling obesity: the role of the NHS in a whole-system approach. *The King's Fund* 2021

For peer review only

Supplementary File 1

Survey

Under the provisions of the Freedom of Information Act 2000, we request the following information:

What is the name of your acute trust?Click or tap here to enter text.

(1) Does your trust provide a weight management service for children living with obesity?

Yes ☐ No ☐

If No, you need not answer further.

If yes, what is the name of the service and where delivered:Click or tap here to enter text.

Who leads this service?Click or tap here to enter text.

It is a hospital ☐ or community-based☐ clinic

(2) What if any are the criteria for eligibility to attend (for instance BMI >98th percentile)?

None ☐

Eligible if: Click or tap here to enter text.

(3) Through what mechanism is the service funded?

CCG ☐

NHS England ☐

Research ☐

Charity ☐

Trust Self-Funding ☐

Other ☐

Please specify Click or tap here to enter text.

(4) Who are the key personnel providing service? Please tick all who apply:

a. Paediatricians ☐
b. Dieticians ☐
c. Psychologists ☐
d. Trained volunteers ☐
e. Hospital or Community based Nursing staff ☐
f. Exercise specialists ☐
g. Social workers ☐
h. CAHMS ☐
i. Youth workers ☐

For peer review only

(5) How many new cases are seen each year? Click or tap here to enter text.

(6) Over how many weeks is the clinic intervention delivered? Click or tap here to enter text.

(7) What is the usual follow up period (entry to discharge?) Click or tap here to enter text.

(8) What final outcomes are collected: Examples: change in BMI, BMI SDS, Self Esteem, Waist Circumference, recruitment, retention.

Please list all. Click or tap here to enter text.

Many thanks for the information you provide.

Professor Julian Hamilton-Shield, University of Bristol

Supplementary File 2

Supplementary Table 1: Service provision according to catchment area characteristics of acute NHS trust

	Mean % of patients within catchment area of acute NHS trusts according to ethnicity and service provision					Mean IMD score of acute NHS trusts
	White	Black	Asian	Mixed	Other	
Service provided	85.5	3.9	7.3	2.2	1.1	22.8
Service not provided	88.6	2.4	6.3	1.9	0.7	20.8
Did not respond to survey	81.7	4.7	9.7	2.6	1.3	22.4

Ethnicity was available for 142 trusts and IMD score for 127 trusts

For peer review

BMJ Open

A cross-sectional survey of child weight management service provision by acute NHS trusts across England in 2020/21

Journal:	*BMJ Open*
Manuscript ID	bmjopen-2022-061971.R1
Article Type:	Original research
Date Submitted by the Author:	18-Aug-2022
Complete List of Authors:	Mears, Ruth; University of Bristol, Centre for Academic Primary Care, Bristol Medical School; University of Bristol, Centre for Exercise Nutrition and Health Sciences<br>Leadbetter, S; University Hospitals Bristol and Weston NHS Foundation Trust, National Institute for Health Research Bristol Biomedical Research Centre; University of Bristol, Faculty of Health Sciences<br>Candler, Toby; University Hospitals Bristol and Weston NHS Foundation Trust, National Institute for Health Research Bristol Biomedical Research Centre; University of Bristol, Faculty of Health Sciences<br>Sutton, Hannah ; University of Bristol, Faculty of Health Sciences<br>Sharp, Deborah; University of Bristol, Centre for Academic Primary Care, Bristol Medical School<br>Shield, Julian; University Hospitals Bristol and Weston NHS Foundation Trust, National Institute for Health Research Bristol Biomedical Research Centre
**Primary Subject Heading**:	Paediatrics
Secondary Subject Heading:	Health services research, Nutrition and metabolism, Public health
Keywords:	PUBLIC HEALTH, PAEDIATRICS, Health policy < HEALTH SERVICES ADMINISTRATION & MANAGEMENT, Organisation of health services < HEALTH SERVICES ADMINISTRATION & MANAGEMENT

SCHOLARONE™
Manuscripts

I, the Submitting Author has the right to grant and does grant on behalf of all authors of the Work (as defined in the below author licence), an exclusive licence and/or a non-exclusive licence for contributions from authors who are: i) UK Crown employees; ii) where BMJ has agreed a CC-BY licence shall apply, and/or iii) in accordance with the terms applicable for US Federal Government officers or employees acting as part of their official duties; on a worldwide, perpetual, irrevocable, royalty-free basis to BMJ Publishing Group Ltd ("BMJ") its licensees and where the relevant Journal is co-owned by BMJ to the co-owners of the Journal, to publish the Work in this journal and any other BMJ products and to exploit all rights, as set out in our licence.

The Submitting Author accepts and understands that any supply made under these terms is made by BMJ to the Submitting Author unless you are acting as an employee on behalf of your employer or a postgraduate student of an affiliated institution which is paying any applicable article publishing charge ("APC") for Open Access articles. Where the Submitting Author wishes to make the Work available on an Open Access basis (and intends to pay the relevant APC), the terms of reuse of such Open Access shall be governed by a Creative Commons licence – details of these licences and which Creative Commons licence will apply to this Work are set out in our licence referred to above.

Other than as permitted in any relevant BMJ Author's Self Archiving Policies, I confirm this Work has not been accepted for publication elsewhere, is not being considered for publication elsewhere and does not duplicate material already published. I confirm all authors consent to publication of this Work and authorise the granting of this licence.

For peer review only

A cross-sectional survey of child weight management service provision by acute NHS trusts across England in 2020/21

Mears R [1,2], Leadbetter S [3,4], Candler T [3,4], Sutton H [4], Sharp D [1], Hamilton-Shield J [3,4].

Centre for Academic Primary Care, Bristol Medical School, University of Bristol, BS8 2PS, UK [1]. Centre for Exercise Nutrition and Health Sciences, School for Policy Studies, University of Bristol, BS8 1TZ, UK [2]. NIHR Bristol Biomedical Research Centre, Nutrition Theme, University of Bristol, BS1 2NT, UK [3]. Faculty of Health Sciences, University of Bristol, BS2 8DZ, UK [4].

Corresponding Author: Dr Ruth Mears, rm14101@bristol.ac.uk

Word Count: 2350

For peer review only - http://bmjopen.bmj.com/site/about/guidelines.xhtml

Abstract

Objective: With one in five children in England living with obesity, we mapped the geographical distribution and format of child weight management services provided by acute NHS trusts across England, to identify breadth of service provision.

Design: A cross-sectional survey.

Setting: The survey was sent to acute NHS trusts (n=148) in England in 2020, via a Freedom of Information (FOI) request.

Participants: Responses were received from 139 of 148 (94%) acute NHS trusts, between March 2020 to March 2021.

Outcome Measures: The survey asked each acute NHS trust whether they provide a weight management service for children living with obesity. For those trusts providing a service, data were collected on eligibility criteria, funding source, personnel involved, number of new patients seen per year, intervention duration, follow-up length and outcome measures. Service characteristics were reported using descriptive statistics. Service provision was analysed in the context of ethnicity and IMD score of the trust catchment area.

Results*:* From the 139 survey respondents, 23% stated that they provided a weight management service for children living with obesity. There were inequalities in the proportion of acute NHS trusts providing a service across the different regions of England, ranging from 4% (Midlands) to 36% (London). For trusts providing a service, there was variability in the number of new cases seen per year, eligibility criteria, funding source, intervention format and outcome measures collected. A multi-disciplinary approach was not routinely provided, with only 41% of services reporting ≥3 different staff disciplines.

Conclusion: In 2020/21, there were geographical inequalities in weight management service provision by acute NHS trusts for children living with obesity. Services provided lacked standardisation, did not routinely offer children multidisciplinary care and were insufficient in size to meet need.

For peer review only - http://bmjopen.bmj.com/site/about/guidelines.xhtml

Strengths and Limitations

- There are no previous studies in the research literature exploring the geographical equity and format of child weight management services provided by acute NHS trusts across England.
- The survey response rate (94%) was good, allowing for an accurate oversight of service provision by acute NHS trusts in England.
- Outcome data for services were not collected, meaning service effectiveness could not be assessed.
- Ethnicity and measures of deprivation data for the individuals accessing the services within each acute NHS trust were not collected, thereby limiting ethnicity and deprivation comparisons to acute NHS trust catchment area level data.

For peer review only

Introduction

Obesity in childhood and adolescence represents a major public health challenge with short and long-term consequences at both an individual and societal level. Children with obesity are more likely to have type 2 diabetes, dyslipidaemia, hypertension, metabolic syndrome, non-alcoholic fatty liver disease and obstructive sleep apnoea [1 2]. Around 55% of children with obesity will remain obese as adolescents, and 80% of adolescents with obesity will continue to be obese as adults [3]. Obesity has both direct healthcare related costs and indirect costs to society through short and long-term inability to work [4 5]. In 2018, obesity cost the UK an estimated £60 billion (3% of its GDP) [6 7].

In the UK, child weight management services are defined as: tier 1 (obesity prevention), tier 2 (lifestyle weight management services), tier 3 (specialist obesity services) and tier 4 (pharmacological or surgical obesity services) [8]. Tier 1 and tier 2 services are commissioned by Public Health working within Local Authorities. In theory, tier 3 and tier 4 services cater for children with severe or complex obesity, or special needs, and are commissioned by Clinical Commissioning Groups (CCGs) working within the National Health Service (NHS). There is no mandatory requirement for Local Authorities or the NHS to commission child weight management services.

Commissioning guidance for specialist services for children with severe or complex obesity, recommend a multi-disciplinary team (MDT) approach, including a paediatrician, dietitian, nurse, physiotherapist, psychologist, liaison psychiatrist and MDT access to a social worker [9]. Previous studies have explored the nature and provision of tier 2 weight management services across England, but there is no documentation within the research literature to gain national oversight of specialist obesity weight management services provided by acute NHS trusts in England [10 11]. A Public Health England (PHE) mapping study of tier 3 weight management services for children in 2015 reported a poor response rate limiting accurate representation of service provision at the time [11]. The lack of understanding about current provision of specialist obesity services was highlighted in the Chief Medical Officer's (CMO) report on childhood obesity [7].

Our local centre has been approached by many teams across England and Wales seeking consultations with our service as local services were not commissioned, suggesting that

For peer review only - http://bmjopen.bmj.com/site/about/guidelines.xhtml

there is no equity of access across the country. The primary objective of the study was to determine how many acute NHS trusts provide a weight management service for children, and the geographical distribution of these services. The secondary objective of the study was to understand the nature of weight management services provided by acute NHS trusts, in terms of eligibility criteria, funding source, whether a MDT approach operated, the reach of the service and details regarding the intervention and outcome data collected. The final objective was to understand whether there were any inequalities in ethnicity and deprivation scores between the catchment populations of NHS trusts providing a weight management service, compared to those that did not.

Methods

Design of the survey

The survey comprised eight questions and was developed by JHS based on his experience working within a paediatric obesity specialist clinic at the Bristol Royal Hospital for Children (BRHC), University Hospitals Bristol & Weston (UHBW) NHS Foundation Trust (Supplementary File 1). All acute NHS trusts in England were asked, 'Does your trust provide a weight management service for children living with obesity?'. Those affirming they did, were asked questions regarding the service's eligibility criteria, source of funding, key personnel involved, number of new patients seen per year, duration of their intervention, length of follow up and outcome data collected by their service. Questions were either free-text or multiple choice (single or multiple answer questions).

Recruitment and characteristics of acute NHS trust participants

The survey was sent by email to each acute NHS trust Freedom Of Information (FOI) office across England (n=148) as a FOI request with a fillable word document for ease of use, to be emailed back to the research team. Where a response was not received by the acute NHS trust within 20 working days, a further reminder email was sent. If no response was received, JHS contacted potential service leads from a list provided by the Children and Young People (CYP) Transformation Programme Team at NHS England.

For peer review only - http://bmjopen.bmj.com/site/about/guidelines.xhtml

The ‘PHE NHS acute trust catchment population tool’ was used to obtain data (where available) about the catchment population for each acute NHS trust, in terms of Index of Multiple Deprivation (IMD) score and ethnicity [12]. This tool also provided details regarding the acute NHS trust type (small, medium, large, multi-service, specialist and teaching).

Data analysis

Data were collated and analysed in Excel version 2206 and Stata version 17. In order to understand potential inequalities in the geographical distribution of weight management services for children, trusts were organised into the seven regions of England (North East and Yorkshire, North West, South East, South West, London, East of England, and Midlands) and the proportion of trusts within each geographical region providing a service was quantified. The mean IMD score and mean proportion of patients within each different ethnicity category were compared between those trusts providing and not providing a service. Comparisons between the proportion of trusts offering and not offering a service were also made according to acute NHS trust type. For acute NHS trusts providing a weight management service, the characteristics of the service provided were reported using descriptive statistics.

Patient and public involvement

Patients or the public were not involved in the design, or conduct, or reporting, or dissemination plans of our research.

For peer review only - http://bmjopen.bmj.com/site/about/guidelines.xhtml

Results

Responses were received between March 2020 and March 2021, from 139 of 148 acute NHS trusts contacted (94%). From the 139 respondents, 32 trusts (23%) stated that they provide a weight management service for children living with obesity. Some trusts offered more than one service for children living with obesity. The mean IMD score, and ethnicity of patients were similar between those within a catchment area of an acute NHS trust providing and not providing a weight management service (Supplementary File 2).

Location in England and acute NHS trust type

The greatest proportion of trusts providing a weight management service were within London (36% of trusts). In contrast, only 4% of trusts in the Midlands provided a service (Table 1). Multi-service and teaching trusts were more likely to provide a weight management service than other acute NHS trusts (Table 2).

For peer review only

Table 1: Geographical distribution of services across England

	Service provided (n=number of acute NHS trusts)	No service provided (n=number of acute NHS trusts)	No response to survey (n=number of acute NHS trusts)
North East and Yorkshire	7 (31.8%)	15 (68.2%)	0 (0%)
North West	4 (17.4%)	17 (73.9%)	2 (8.7%)
South East	4 (21.1%)	15 (78.9%)	0 (0%)
London	9 (36.0%)	14 (56%)	2 (8%)
East of England	3 (16.7%)	14 (77.8%)	1 (5.6%)
Midlands	1 (4.2%)	20 (83.3%)	3 (12.5%)
South West	4 (23.5%)	12 (70.6%)	1 (5.9%)
Total	32 (22%)	107 (72%)	9 (6%)

Table 2: Service provision according to acute NHS trust type

	Service provided (n=number of acute NHS trusts)	No service provided (n=number of acute NHS trusts)	No response to survey (n=number of acute NHS trusts)
Acute – Small	6 (19.4%)	24 (77.4%)	1 (3.2%)
Acute – Medium	6 (19.4%)	23 (74.2%)	2 (6.5%)

For peer review only - http://bmjopen.bmj.com/site/about/guidelines.xhtml

Acute – Large	5 (18.5%)	20 (74.1%)	2 (7.4%)
Acute - Multi-Service	2 (66.7%)	1 (33.3%)	0 (0%)
Acute – Specialist	3 (18.8%)	13 (81.3%)	0 (0%)
Acute – Teaching	10 (29.4%)	22 (64.7%)	2 (5.9%)
Total	32 (23%)	103 (73%)	7 (5%)
Data regarding the type of acute NHS trust was available for 142 trusts			

Site of intervention, eligibility criteria, funding source and staffing

The characteristics of weight management services provided by acute NHS trusts across England, is summarised in Table 3. Over half of weight management services were hospital based only (59%). There was considerable variation in the source of funding for these services, with 26% of trusts receiving funding from multiple sources. CCGs were the most frequently cited source of funding.

Eligibility criteria for services were mainly based around a measure of weight (e.g., Body Mass Index [BMI], BMI percentile, BMI z-score) but some services reported less specific eligibility criteria (e.g. 'all referrals where a dietetic or nutrition related problem has been identified by a medic'). Dietitians were the most frequently reported personnel involved in service provision, followed by paediatricians. Most services did not provide a multi-disciplinary team approach, with 31% of services reporting only one type of staffing personnel involved.

Table 3: Characteristics of weight management services provided by acute NHS trusts

	**Number of acute NHS trusts (n=32)**
**Location of services provided by each acute NHS trust**	
Hospital	19
Community	8
Hospital and community-based	4
Not specified	1
**Source of Funding ***	
Clinical Commissioning Group (CCG)	15
Trust Self-Funding	9
NHS England	2
County Council, Local Authority or Public Health	7
Other	7
Not specified	1

For peer review only - http://bmjopen.bmj.com/site/about/guidelines.xhtml

**Eligibility criteria ****	
BMI >91st centile	5
BMI >91st centile and another criterion	5
BMI >98th centile	5
BMI >98th centile and another criterion	9
BMI >99.6th centile	4
BMI >99.6th centile and another criterion	5
BMI Standard Deviation Score (SDS) >2.2	1
BMI ≥3.33sd in child over 2 years old	1
BMI>+3.5SDS or BMI> +3.0SDS with comorbidities	2
Other	18
**Staff disciplines involved in service provision for each acute NHS trust *****	
Dietitians	26
Paediatricians	16
Hospital or community-based nursing staff	11
Psychologists	6
Exercise specialists	5
Children and Adolescent Mental Health Services (CAMHS)	3
Social workers	2
Not specified	2
Trained volunteers	1
**Number of staff disciplines providing service for each acute NHS trust**	
One	10
Two	7
Three	6
Four	5
Five	1
Six	1
Not specified	2

* Some acute NHS trusts received funding from more than one source for their service(s).
** Some acute NHS trusts reported more than one different eligibility criteria for their service(s).
*** Some acute NHS trusts reported more than one different staff disciplines involved in their service provision.

Characteristics of services provided by acute NHS trusts

Table 4 summarises details of the interventions provided by trusts. There was variation in the number of new cases seen per year, with 57% of acute NHS trusts reporting that their

For peer review only - http://bmjopen.bmj.com/site/about/guidelines.xhtml

service(s) saw ≤100 new cases per year. Many trusts reported that the intervention and follow-up period were tailored to the individuals needs rather than following a pre-specified pattern. Outcome measures most frequently collected were BMI, BMI z-score / SDS or BMI centile. A variety of other outcome measures were reported, including a parent or user satisfaction survey, changes to emotional eating behaviours, reduction of inequalities among targeted groups and SCORE-15 index of family functioning and change [13].

Table 4: Details of the services provided by acute NHS trusts

	**Number of acute NHS trusts (n=32)**
**Total number of new cases seen per year for each acute NHS trust**	
≤ 50	11
51-100	8
101-150	4
151-200	2
201-250	0
251-300	0
301-350	2
Not specified	5
**Length of intervention***	
0-6 months	9
6-12 months	4
12-18 months	1
Tailored to the individual	11
Not clearly specified	10
Other	2
**Usual follow-up period***	
0-6 months	6
6-12 months	5
12-18 months	6
over 18 months	4
Tailored to the individual	9
Not clearly specified	3
Other	1
**Outcome Measures**	
BMI	19
BMI-SDS or BMI z-score	11
BMI-centile	2
Weight	7

For peer review only - http://bmjopen.bmj.com/site/about/guidelines.xhtml

Waist circumference	2
Body composition	3
Blood pressure	2
Sleep study results	3
Blood tests	4
Imaging	1
Changes to diet	7
Changes to physical activity levels	4
Changes to mental wellbeing	7
Quality of life	2
Other	12
**Respondents sometimes detailed more than one clinical service in the trust so n responses may be greater than 32.*	

Discussion

Main Findings

There were significant gaps in the provision of child weight management services by acute NHS trusts across England, with 77% of survey respondents not providing a service. Within England, there was considerable variation in the distribution of services with a greater proportion of acute NHS trusts providing a service in certain geographical regions (e.g., London 36%, and the North-East and Yorkshire 32%) compared to others (e.g., Midlands 4%). There was a lack of consistency in the funding source and eligibility criteria for children to access services between different acute NHS trusts. A multi-disciplinary approach was not routinely provided and there was no standardisation of the service provided between different trusts in terms of length of intervention, follow-up period and outcome measures.

Strengths and Weaknesses

This is the first study in the research literature to explore the nature of child weight management service provision by acute NHS trusts across England. The response rate by acute NHS trusts was good (94%). As acute NHS trusts are typically responsible for the provision of tier 3 weight management services, this paper provides a good indicator of what services are available for children with severe obesity. It was not within the remit of this study to capture non-NHS tier 2 weight management programmes. It was also not

For peer review only - http://bmjopen.bmj.com/site/about/guidelines.xhtml

possible to merge the data on NHS provision with pre-existing datasets about non-NHS tier 2 weight management provision (i.e. those commissioned by Local Authorities), partly due to the rapid commissioning and de-commissioning of the latter [10] .

Data detailing intervention components and outcome measures were not requested to reduce burden on individual Trust's FOI response teams and improve overall response rate [14]. Thus, it was not possible to assess the relative effectiveness of different interventions offered by each acute NHS trust. However, given the lack of standardization in eligibility criteria and outcome measures across different NHS trusts, meaningful comparative analyses are unlikely to have been possible. Deprivation and ethnicity data for those actually accessing each service were not collected, so comparisons in these measures were limited to acute NHS trust population catchment area data. The newly commissioned 'Complications from Excess Weight' (CEW) clinics are collecting robust process and outcome data as a national audit and we envisage that all child weight management services will be encouraged to report similar data in the future [15].

Data were collected through a FOI request meaning it is not possible to ascertain who supplied survey data for each NHS trust. The assumption is that the FOI request would have been appropriately handed to the lead for the clinical service.

Contextualisation and Implications of Findings

A lack of adequate multidisciplinary weight management clinics for children with obesity and unequal geographical distribution of available services, has also been described in Australia [16 17]. This contrasts with current guidelines and expert recommendations for obesity management in children, which advises a multidisciplinary team approach to ensure optimal care [9 18 19]. Similar to our study, data reported from Canadian paediatric weight management services has shown variability in eligibility criteria, clinic size and intervention length [20].

In England (2020/21), 14% of children aged 4-5 years old and 26% of children aged 10-11 years old were identified as obese by the National Child Measurement Programme (NCMP) [21]. Therefore, the current 32 sparsely distributed acute NHS trust services in England

For peer review only - http://bmjopen.bmj.com/site/about/guidelines.xhtml

(generally seeing <200 new cases per year), will only reach a minute fraction of children living with obesity (or severe obesity).

A pathway modelling study of National Institute for Health and Care Excellence (NICE) guidance against Health Survey for England data, suggested that there is a ‘need for consistent evidence-based commissioning of services across the childhood obesity pathway based on population burden’, however our study indicates this is still not happening [22]. Despite the West Midlands having the highest prevalence of severe obesity according to 2019/2020 reception NCMP data (3.1% vs England average of 2.5%), the Midlands region had the lowest proportion of NHS trusts providing a weight management service (n=1/24, 4.2%) [21].

The NHS Long Term Plan aims to treat a further 1000 children a year for severe complications related to their obesity through ‘Complications from Excess Weight’ (CEW) clinics. These CEW clinics have a universal data collection system for patient demographics and outcomes to allow comparison across sites and understand the reach of the service according to ethnicity and deprivation. However with previous estimates from 2014 suggesting that 556,000 children would be eligible for assessment in secondary care, this planned expansion of services will still not reach the majority of children who may require treatment [22 23].

The lack of consistent sources of funding for specialist obesity clinics in acute NHS trusts likely contributes to the inequalities in the geographical distribution of services. Clearly defined routes of funding for child weight management clinics in acute NHS trusts are needed to help minimise inequalities in service provision. Furthermore, given the 13 percentage-point gap in obesity rates between the most deprived and least deprived children in 2019, services need to ensure that within the geographical areas they provide for, they are reaching the most deprived children [24].

Conclusion

There needs to be a clear, realistic national strategy outlining who should receive initial priority for specialist multi-disciplinary obesity care, as the need for support will likely

For peer review only - http://bmjopen.bmj.com/site/about/guidelines.xhtml

exceed capacity for the foreseeable future and service provision across England should be equitable. The CEW clinics may help to reduce current inequalities in service provision and target those at greatest risk from obesity. However, significant gaps in support for children who are obese but do not meet the CEW clinic criteria will likely remain, especially given the variable tier 2 service provision across England [10]. Furthermore, as referral into the CEW clinic will be reliant on the presence of co-morbidities, consideration needs to be given to determine the most appropriate way of ensuring that children living with obesity (or at least severe obesity) are assessed for co-morbidities and thereby given the opportunity to access specialist care where needed.

Funding: Dr Ruth Mears is funded by a National Institute for Health Research (NIHR) In-Practice Fellowship Award (NIHR-IPF-16-10-07) for this research. The NIHR Biomedical Research Centre at University Hospitals Bristol and Weston NHS Foundation Trust supports Professor Julian Hamilton-Shield.

Disclaimer: This publication presents independent research funded by the National Institute for Health Research (NIHR). The views expressed in this publication are those of the authors and not necessarily those of the NIHR.

Competing Interests: JHS is clinical lead for a Tier 3 childhood obesity service in Bristol and will be a hub for the new 'Complications from Excess Weight' (CEW) clinics funded by NHS England.

Contributors: JHS designed the study. TC, SL and HS collected the data. RM and JHS analysed the data and drafted the initial manuscript. JHS, TC, SL, HS and DS revised the final manuscript.

Data sharing: All data relevant to the study are included in the article or uploaded as supplementary information.

Ethical approval

This is a health services research paper. The survey was sent to acute NHS trusts as a FOI request, therefore Ethics Committee review was not required.

For peer review only - http://bmjopen.bmj.com/site/about/guidelines.xhtml

References

1. Sharma V, Coleman S, Nixon J, et al. A systematic review and meta-analysis estimating the population prevalence of comorbidities in children and adolescents aged 5 to 18 years. *Obes Rev* 2019;20(10):1341-49. doi: 10.1111/obr.12904 [published Online First: 2019/07/26]
2. Li AM, So HK, Au CT, et al. Epidemiology of obstructive sleep apnoea syndrome in Chinese children: a two-phase community study. *Thorax* 2010;65(11):991-7. doi: 10.1136/thx.2010.134858 [published Online First: 2010/10/23]
3. Simmonds M, Llewellyn A, Owen CG, et al. Predicting adult obesity from childhood obesity: a systematic review and meta-analysis. *Obes Rev* 2016;17(2):95-107. doi: 10.1111/obr.12334 [published Online First: 2015/12/24]
4. Wang YC, McPherson K, Marsh T, et al. Health and economic burden of the projected obesity trends in the USA and the UK. *Lancet* 2011;378(9793):815-25. doi: 10.1016/S0140-6736(11)60814-3 [published Online First: 2011/08/30]
5. Goettler A, Grosse A, Sonntag D. Productivity loss due to overweight and obesity: a systematic review of indirect costs. *BMJ Open* 2017;7(10):e014632. doi: 10.1136/bmjopen-2016-014632
6. Mckinsey Global Institute. Overcoming obesity: An initial economic analysis. 2014. https://www.mckinsey.com/~/media/mckinsey/business%20functions/economic%20studies%20temp/our%20insights/how%20the%20world%20could%20better%20fight%20obesity/mgi_overcoming_obesity_full_report.ashx [Accessed July 2022]
7. Davies PDS. Time to Solve Childhood Obesity. An Independent Report by the Chief Medical Officer, 2019.
8. The National Institute for Health and Care Excellence (NICE). Weight management: lifestyle services for overweight or obese children and young people. PH47 https://www.nice.org.uk/guidance/ph53 [Accessed August 2021].
9. Welbourn R, Hopkins J, Dixon JB, et al. Commissioning guidance for weight assessment and management in adults and children with severe complex obesity. *Obesity Reviews* 2018;19(1):14-27. doi: 10.1111/obr.12601
10. Mears R, Jago R, Sharp D, et al. Exploring how lifestyle weight management programmes for children are commissioned and evaluated in England: a mixed methodology study. *BMJ Open* 2019;9(12):e025423. doi: 10.1136/bmjopen-2018-025423 [published Online First: 2019/12/19]
11. Public Health England (PHE). National mapping of weight management services. Provision of Tier 2 and Tier 3 services in England: Public Health England, 2015.
12. Public Health England (PHE). NHS Acute Trust Catchment Populations Tool https://app.powerbi.com/view?r=eyJrIjoiODZmNGQ0YzItZDAwZi00MzFiLWE4NzAtMzVmNTUwMThmMTVlIiwidCI6ImVlNGUxNDk5LTRhMzUtNGIyZS1hZDQ3LTVmM2NmOWRlODY2NiIsImMiOjh92020 [Accessed October 2021]
13. Stratton P, Lask J, Bland J, et al. Detecting therapeutic improvement early in therapy: Validation of the SCORE-15 index of family functioning and change. *Journal of Family Therapy* 2014;36(1):3-19. doi: 10.1111/1467-6427.12022
14. University College London (UCL). Making Freedom of Information Requests: A Guide for Academic Researchers. The Constitution Unit 2012 [Accessed February 2022] https://www.ucl.ac.uk/constitution-unit/sites/constitution_unit/files/academics-guide-to-foi.pdf.
15. National Health Service (NHS). Complications from Excess Weight (CEW) clinics for children https://www.england.nhs.uk/get-involved/cyp/specialist-clinics-for-children-and-young-people-living-with-obesity/ [Accessed July 2022].
16. McMaster CM, Calleja E, Cohen J, et al. Current status of multi-disciplinary paediatric weight management services in Australia. *J Paediatr Child Health* 2021;57(8):1259-66. doi: 10.1111/jpc.15439 [published Online First: 2021/03/17]

For peer review only - http://bmjopen.bmj.com/site/about/guidelines.xhtml

17. Spilchak PJ, Denney-Wilson E, King L, et al. Tertiary paediatric obesity services in Australia. *J Paediatr Child Health* 2008;44(5):243-7. doi: 10.1111/j.1440-1754.2007.01266.x [published Online First: 2008/01/16]
18. Styne DM, Arslanian SA, Connor EL, et al. Pediatric Obesity-Assessment, Treatment, and Prevention: An Endocrine Society Clinical Practice Guideline. *J Clin Endocrinol Metab* 2017;102(3):709-57. doi: 10.1210/jc.2016-2573 [published Online First: 2017/03/31]
19. Barlow SE. Expert committee recommendations regarding the prevention, assessment, and treatment of child and adolescent overweight and obesity: summary report. *Pediatrics* 2007;120 Suppl 4:S164-92. doi: 10.1542/peds.2007-2329C [published Online First: 2007/12/18]
20. Zahn K, Ibrahim Q, Ball GDC, et al. Variability in How Canadian Pediatric Weight Management Clinics Deliver Care: Evidence from the CANadian Pediatric Weight Management Registry. *Child Obes* 2021;17(6):420-26. doi: 10.1089/chi.2021.0011 [published Online First: 2021/05/13]
21. Public Health England (PHE). Obesity Profile PHE Fingertips NCMP data https://fingertips.phe.org.uk/profile/national-child-measurement-programme/data#page/0/gid/8000011/ati/6/iid/90316/age/200/sex/4/cat/-1/ctp/-1/cid/4/tbm/1 [Accessed August 2021]
22. Viner RM, Kinra S, Nicholls D, et al. Burden of child and adolescent obesity on health services in England. *Arch Dis Child* 2018;103(3):247-54. doi: 10.1136/archdischild-2017-313009 [published Online First: 2017/08/03]
23. National Health Service (NHS). NHS Long Term Plan. Chapter 2: More NHS action on prevention and health inequalities
24. Holmes J. Tackling obesity: the role of the NHS in a whole-system approach. *The King's Fund* 2021

For peer review only

For peer review only - http://bmjopen.bmj.com/site/about/guidelines.xhtml

Supplementary File 1

Survey

Under the provisions of the Freedom of Information Act 2000, we request the following information:

What is the name of your acute trust?Click or tap here to enter text.

(1) Does your trust provide a weight management service for children living with obesity?

Yes ☐ No ☐

If No, you need not answer further.

If yes, what is the name of the service and where delivered:Click or tap here to enter text.

Who leads this service?Click or tap here to enter text.

It is a hospital ☐ or community-based☐ clinic

(2) What if any are the criteria for eligibility to attend (for instance BMI >98th percentile)?

None ☐

Eligible if: Click or tap here to enter text.

(3) Through what mechanism is the service funded?

CCG ☐

NHS England ☐

Research ☐

Charity ☐

Trust Self-Funding ☐

Other ☐

Please specify Click or tap here to enter text.

(4) Who are the key personnel providing service? Please tick all who apply:

a. Paediatricians ☐
b. Dieticians ☐
c. Psychologists ☐
d. Trained volunteers ☐
e. Hospital or Community based Nursing staff ☐
f. Exercise specialists ☐
g. Social workers ☐
h. CAHMS ☐
i. Youth workers ☐

For peer review only

(5) How many new cases are seen each year? Click or tap here to enter text.

(6) Over how many weeks is the clinic intervention delivered? Click or tap here to enter text.

(7) What is the usual follow up period (entry to discharge?) Click or tap here to enter text.

(8) What final outcomes are collected: Examples: change in BMI, BMI SDS, Self Esteem, Waist Circumference, recruitment, retention.

Please list all. Click or tap here to enter text.

Many thanks for the information you provide.

Professor Julian Hamilton-Shield, University of Bristol

Supplementary File 2

Supplementary Table 1: Service provision according to catchment area characteristics of acute NHS trust

	Mean % of patients within catchment area of acute NHS trusts according to ethnicity and service provision					Mean IMD score of acute NHS trusts
	White	Black	Asian	Mixed	Other	
Service provided	85.5	3.9	7.3	2.2	1.1	22.8
Service not provided	88.6	2.4	6.3	1.9	0.7	20.8
Did not respond to survey	81.7	4.7	9.7	2.6	1.3	22.4

Ethnicity was available for 142 trusts and IMD score for 127 trusts

For peer review only - http://bmjopen.bmj.com/site/about/guidelines.xhtml

The RECORD statement – checklist of items, extended from the STROBE statement, that should be reported in observational studies using routinely collected health data.

	Item No.	STROBE items	Location in manuscript where items are reported	RECORD items	Location in manuscript where items are reported
**Title and abstract**					
	1	(a) Indicate the study's design with a commonly used term in the title or the abstract (b) Provide in the abstract an informative and balanced summary of what was done and what was found	**Page 1, line 1-2:** The title of the paper includes the study design (cross-sectional survey).<br><br>**Page 2, line 25-50:** The abstract summarises what was done (survey via FOI request) and what was found.	RECORD 1.1: The type of data used should be specified in the title or abstract. When possible, the name of the databases used should be included.<br><br>RECORD 1.2: If applicable, the geographic region and timeframe within which the study took place should be reported in the title or abstract.<br><br>RECORD 1.3: If linkage between databases was conducted for the study, this should be clearly stated in the title or abstract.	**Page 1 and 2:** The title and abstract specify the type of data used (i.e. survey data from a FOI request), the geographic region (i.e. England) and the timeframe (i.e. March 2020 to March 2021) the study took place. No linkage between databases was conducted for the study.
**Introduction**					
Background rationale	2	Explain the scientific background and rationale for the investigation being reported	**Page 4 and 5, line 78-107**		
Objectives	3	State specific objectives, including any prespecified hypotheses	**Page 5, line 107 to 115**		
Methods					
Study Design	4	Present key elements of study design early in the paper	**Page 5-6, line 118-138**		
Setting	5	Describe the setting, locations, and relevant dates, including	**Page 5-6, line 128-138**		

		periods of recruitment, exposure, follow-up, and data collection			
Participants	6	*(a) Cohort study* - Give the eligibility criteria, and the sources and methods of selection of participants. Describe methods of follow-up<br>*Case-control study* - Give the eligibility criteria, and the sources and methods of case ascertainment and control selection. Give the rationale for the choice of cases and controls<br>*Cross-sectional study* - Give the eligibility criteria, and the sources and methods of selection of participants<br><br>*(b) Cohort study* - For matched studies, give matching criteria and number of exposed and unexposed<br>*Case-control study* - For matched studies, give matching criteria and the number of controls per case	**Page 5-6, line 128-138**	RECORD 6.1: The methods of study population selection (such as codes or algorithms used to identify subjects) should be listed in detail. If this is not possible, an explanation should be provided.<br><br>RECORD 6.2: Any validation studies of the codes or algorithms used to select the population should be referenced. If validation was conducted for this study and not published elsewhere, detailed methods and results should be provided.<br><br>RECORD 6.3: If the study involved linkage of databases, consider use of a flow diagram or other graphical display to demonstrate the data linkage process, including the number of individuals with linked data at each stage.	**Page 5-6, line 129-138** details the methods of study population selection (i.e. each acute NHS trust in England).
Variables	7	Clearly define all outcomes, exposures, predictors, potential confounders, and effect modifiers. Give diagnostic criteria, if applicable.	**Page 5, line 119 – 127 & Supplementary File 1**	RECORD 7.1: A complete list of codes and algorithms used to classify exposures, outcomes, confounders, and effect modifiers should be provided. If these cannot be reported, an explanation should be provided.	**Supplementary File 1 & page 6, line pg 139-150.**
Data sources/ measurement	8	For each variable of interest, give sources of data and details of methods of assessment (measurement).	**Page 5-6, line 130-142, line 143-153 & Supplementary File 1 &**		

For peer review only

		Describe comparability of assessment methods if there is more than one group			
Bias	9	Describe any efforts to address potential sources of bias	**NA – the survey was sent as a FOI request.**		
Study size	10	Explain how the study size was arrived at	**Page 5, line 118-127.**		
Quantitative variables	11	Explain how quantitative variables were handled in the analyses. If applicable, describe which groupings were chosen, and why	**Page 6, line 139-150**		
Statistical methods	12	(a) Describe all statistical methods, including those used to control for confounding<br>(b) Describe any methods used to examine subgroups and interactions<br>(c) Explain how missing data were addressed<br>(d) *Cohort study* - If applicable, explain how loss to follow-up was addressed<br>*Case-control study* - If applicable, explain how matching of cases and controls was addressed<br>*Cross-sectional study* - If applicable, describe analytical methods taking account of sampling strategy<br>(e) Describe any sensitivity analyses	**Page 6, line 139-150**		
Data access and cleaning methods		..	**Page 5, line 128-134**	RECORD 12.1: Authors should describe the extent to which the investigators had access to the database	**Supplementary File 1**

For peer review only

				population used to create the study population.<br><br>RECORD 12.2: Authors should provide information on the data cleaning methods used in the study.	
Linkage		..	**N/a – no linkage**	RECORD 12.3: State whether the study included person-level, institutional-level, or other data linkage across two or more databases. The methods of linkage and methods of linkage quality evaluation should be provided.	**N/a – no linkage**
**Results**					
Participants	13	(a) Report the numbers of individuals at each stage of the study (*e.g.*, numbers potentially eligible, examined for eligibility, confirmed eligible, included in the study, completing follow-up, and analysed)<br>(b) Give reasons for non-participation at each stage.<br>(c) Consider use of a flow diagram	**Pg 7, line 163-168**	RECORD 13.1: Describe in detail the selection of the persons included in the study (*i.e.,* study population selection) including filtering based on data quality, data availability and linkage. The selection of included persons can be described in the text and/or by means of the study flow diagram.	**Pg 7, line 163-168.**<br><br>**Page 5 & 6, line 129-140**
Descriptive data	14	(a) Give characteristics of study participants (*e.g.*, demographic, clinical, social) and information on exposures and potential confounders<br>(b) Indicate the number of participants with missing data for each variable of interest<br>(c) *Cohort study* - summarise follow-up time (*e.g.*, average and total amount)	**Pg 7 and 8, line 175-178 (Table 1 & Table 2). Supplementary File 2**		

For peer review only

Outcome data	15	*Cohort study* - Report numbers of outcome events or summary measures over time<br>*Case-control study* - Report numbers in each exposure category, or summary measures of exposure<br>*Cross-sectional study* - Report numbers of outcome events or summary measures	**Page 8 to 11, line 179- 204**		
Main results	16	(a) Give unadjusted estimates and, if applicable, confounder-adjusted estimates and their precision (e.g., 95% confidence interval). Make clear which confounders were adjusted for and why they were included<br>(b) Report category boundaries when continuous variables were categorized<br>(c) If relevant, consider translating estimates of relative risk into absolute risk for a meaningful time period	**Page 7 to 11, line 162 to 204**		
Other analyses	17	Report other analyses done—e.g., analyses of subgroups and interactions, and sensitivity analyses	**Supplementary Table 2**		
**Discussion**					
Key results	18	Summarise key results with reference to study objectives	**Page 11, line 207 - 216**		
Limitations	19	Discuss limitations of the study, taking into account sources of potential bias or imprecision. Discuss both direction and magnitude of any potential bias	**Page 11, line 217 - 241**	RECORD 19.1: Discuss the implications of using data that were not created or collected to answer the specific research question(s). Include discussion of misclassification bias, unmeasured confounding, missing data, and changing eligibility over	**Page 11, line 217 - 241**

For peer review only

				time, as they pertain to the study being reported.	
Interpretation	20	Give a cautious overall interpretation of results considering objectives, limitations, multiplicity of analyses, results from similar studies, and other relevant evidence	**Page 12 & 13, line 242 to 290**		
Generalisability	21	Discuss the generalisability (external validity) of the study results	**Page 3, line 57-63. Page 11, line 217-243**		
**Other Information**					
Funding	22	Give the source of funding and the role of the funders for the present study and, if applicable, for the original study on which the present article is based	**Page 14, line 292-295**		
Accessibility of protocol, raw data, and programming code		..	**Page 14, line 305-306**	RECORD 22.1: Authors should provide information on how to access any supplemental information such as the study protocol, raw data, or programming code.	**Page 14, line 305-306**

*Reference: Benchimol EI, Smeeth L, Guttmann A, Harron K, Moher D, Petersen I, Sørensen HT, von Elm E, Langan SM, the RECORD Working Committee. The REporting of studies Conducted using Observational Routinely-collected health Data (RECORD) Statement. *PLoS Medicine* 2015; in press.

*Checklist is protected under Creative Commons Attribution (CC BY) license.

For peer review only
